# Efficacy of Platelet-Rich Plasma (PRP) in Treating Plantar Fasciitis

**DOI:** 10.7759/cureus.72454

**Published:** 2024-10-26

**Authors:** Muhammad Mannan, Faisal Karim, Usman Hafeez, Sarmad Khalil

**Affiliations:** 1 Trauma and Orthopaedics, Sheikh Zayed Medical College and Hospital, Rahim Yar Khan, Rahim Yar Khan, PAK; 2 Orthopaedic Surgery, University Hospitals Birmingham NHS Foundation Trust, Birmingham, GBR; 3 Trauma and Orthopaedics, Ghurki Trust Teaching Hospital, Lahore, PAK; 4 Trauma and Orthopaedics, University Hospitals Birmingham NHS Foundation Trust, Birmingham, GBR; 5 Orthopedics and Traumatology, Queen Elizabeth Hospital Birmingham, Birmingham, GBR

**Keywords:** growth factors, plantar fasciitis, platelet-rich plasma, prp, tissue repair

## Abstract

Background: Platelet-rich plasma (PRP) is an autologous blood derivative with an enhanced platelet concentration and can enhance the tissue-healing process naturally. Plasma with a higher concentration of platelets on activation leads to the release of various growth factors that in turn augment tissue repair and jump-start the healing process. The aim of this study was to determine the efficacy of PRP in reducing pain and improving functional outcomes in patients with plantar fasciitis who had failed to respond to conservative treatment.

Methods: A retrospective study was performed at the Orthopedics Department of the Ghurki Trust Teaching Hospital, Lahore, during March 2020 to January 2021. A total of 140 patients, 25 to 65 years of age, with plantar fasciitis were included. Patients who had undergone foot surgery in the past, those with a generalized inflammatory disorder, pregnant female patients, those taking anticoagulants and those with tumours of the lower extremity were excluded. Following aseptic measures, 2.5 ml of PRP containing 5.5% calcium chloride was administered at the most tender point on the medial aspect of the heel. All injections were administered by the principal researcher himself. All patients were followed at regular intervals post-therapy and efficacy was measured at the end of three months post-therapy.

Results: The average age of participants was 41.56 ± 10.37 years, with most patients (94 out of 140, 67.14%) falling in the 25-45 age range. The study population consisted of 89 male (63.57%) and 51 female (36.43%) patients, with a male-to-female ratio of 1.7:1. PRP therapy was effective in 131 patients (93.57%), and age was found to be a significant factor influencing treatment success (p < 0.05).

Conclusion: PRP is a highly effective treatment option for plantar fasciitis, providing substantial symptomatic relief and improving patient outcomes, specially in patients unresponsive to conservative treatment. Further research is necessary to confirm these results and establish standardized guidelines for its use.

## Introduction

Plantar fasciitis is recognized as the leading cause of heel pain. Although the name suggests inflammation, studies have shown that in the chronic phase, inflammatory cells are absent [1]. As a result, plantar fasciitis is now considered a degenerative condition rather than inflammatory, driven by repetitive microtrauma at the point of insertion, similar to the pathophysiology of lateral epicondylitis of the elbow [2]. The condition typically affects middle-aged and older adults, with a slight predominance in women. Key risk factors include obesity, running, and prolonged standing [3]. Plantar fasciitis is generally self-limiting, with patients commonly reporting medial heel pain, particularly noticeable when getting out of bed in the morning. In persistent cases, the pain can become chronic and constant [4,5]. Although the majority of cases resolve on their own, around 10% can develop into a chronic, long-term issue [6].

Conservative treatment approaches are the first line of management, including shoe orthotics, shockwave therapy, stretching exercises, and analgesics. For more resistant cases, interventions such as steroid injections, botulinum toxin type A, and autologous therapies like platelet-rich plasma (PRP) are employed [3,7]. Surgical options, such as fasciotomy, are reserved for the most challenging cases [8]. While steroid injections can provide temporary pain relief, they come with risks such as plantar fascia rupture and potential infections [9].

PRP has emerged as a promising treatment option due to its ability to deliver growth factors and proteins that enhance tissue healing with minimal side effects [10]. Research indicates that PRP, particularly when platelet counts exceed 1,000,000/μl in 5 ml of plasma, may offer more prolonged pain relief compared to steroid injections [6,7]. However, much of the existing literature is based on Western populations, and there is limited data from regions where steroid use remains prevalent [8,9].

The aim of our study was to assess the efficacy of PRP in treating plantar fasciitis, particularly in terms of pain relief and functional improvement, allowing patients to return to their daily activities more quickly and with fewer complications.

## Materials and methods

A retrospective study was conducted at the Orthopedics Department of the Ghurki Trust Teaching Hospital, Lahore, from July 2020 to January 2021. The sample size was calculated to be 140 patients, with a 95% confidence level, 3% margin of error, and an expected efficacy rate of PRP for treating plantar fasciitis set at 96.67%. Non-probability consecutive sampling was employed to select the participants. Ethical approval was granted by the Institutional Review Board of the Ghurki Trust Teaching Hospital. Written informed consent was obtained from all participants prior to the study.

Inclusion and exclusion criteria

The study included patients between the ages of 25 and 65 years, of both genders, who presented with plantar fasciitis of more than one-month duration and were unresponsive to conservative treatment. Patients were excluded if they had bilateral plantar fasciitis, were pregnant, had undergone foot surgery in the past, or had generalized inflammatory conditions (e.g., gout, ankylosing spondylitis, rheumatoid arthritis, or lupus). Other exclusion criteria included steroid use within the past month, use of anticoagulants (e.g., aspirin, heparin) within the past week, the presence of tumors of the lower extremity, and bleeding disorders (international normalized ratio, or INR > 1.2).

Procedure

Each patient received a single injection of 2.5 ml of PRP, which was prepared by drawing 20 ml of autologous blood. The blood was centrifuged at 1500 rpm for 10 minutes to separate the platelet-rich plasma. The PRP was then activated by adding 5.5% calcium chloride. The injection was administered at the most tender point on the medial aspect of the heel under aseptic conditions by the principal researcher. No ultrasound guidance was used for the injections. Following the procedure, patients were advised to avoid heavy weight-bearing activities for two weeks, but were encouraged to perform gentle stretching exercises.

Data collection and statistical analysis

Data were collected using a structured form and analyzed using IBM SPSS Statistics, version 20.0 (IBM Corp., Armonk, NY). Continuous variables such as age, duration of disease, BMI, and pre-therapy pain scores were expressed as means and standard deviations. Categorical variables, including gender, side affected (right/left), occupation (field/office/domestic), presence of diabetes mellitus, and treatment efficacy, were presented as frequencies and percentages.

Efficacy definition

Efficacy was operationally defined based on the reduction in heel pain and improvement in functional mobility. Pain reduction was measured using the visual analog scale (VAS), and functional improvement was assessed through the Foot and Ankle Ability Measure (FAAM). Patients who demonstrated a ≥50% reduction in pain on the VAS and significant improvement in FAAM scores at the three-month follow-up were considered to have achieved successful treatment outcomes.

Stratification was performed for age, gender, duration of disease, pre-therapy pain score, BMI, the affected side, occupation, and diabetes mellitus. A chi-square test was applied post-stratification to assess the impact of these factors on treatment efficacy. A p-value ≤0.05 was considered statistically significant.

## Results

The age range in this study was 25 to 65 years, with a mean age of 41.56 ± 10.37 years. A majority of patients, 94 (67.14%), were between 25 and 45 years of age. Out of the 140 participants, 89 (63.57%) were male and 51 (36.43%) were female patients, resulting in a male-to-female ratio of approximately 1.7:1. The mean duration of the disease was 4.13 ± 1.41 months, with 80 patients (57.14%) having the disease for three months or less, while 60 patients (42.86%) had it for longer. The mean BMI was 28.91 ± 3.11 kg/m², and a majority of participants, 93 (66.43%), had a BMI greater than 27, indicating a high prevalence of overweight or obesity. The distribution of the affected side was nearly equal, with 72 patients (51.43%) affected on the left side and 68 patients (48.57%) on the right. Regarding diabetes, 56 patients (40.00%) were diabetic, while 84 (60.00%) were not. In this study, the efficacy of platelet-rich plasma for treating plantar fasciitis was observed in 131 patients (93.57%), demonstrating a high success rate. Stratification of the efficacy based on demographic factors revealed that age was the only significant factor influencing the efficacy of PRP treatment for plantar fasciitis (p < 0.05). The demographic details are given in Table 1.

**Table 1:** Demographic distribution of patients

Parameter	Category	Frequency (%)	Mean ± SD
Gender	Male	89 (63.57)	
	Female	51 (36.43)	
Age (years)	25-45	94 (67.14)	41.56 ± 10.37 years
	46-65	46 (32.86)	
Duration of disease	≤3 months	80 (57.14)	4.13 ± 1.41 months
	>3 months	60 (42.86)	
BMI (kg/m²)	≤27	47 (33.57)	28.91 ± 3.11 kg/m²
	>27	93 (66.43)	
Side affected	Left	72 (51.43)	
	Right	68 (48.57)	
Diabetes mellitus	Yes	56 (40.00)	
	No	84 (60.00)	
Efficacy of platelet-rich plasma	Yes	131 (93.57)	
	No	9 (6.43)	

**Figure 1:**
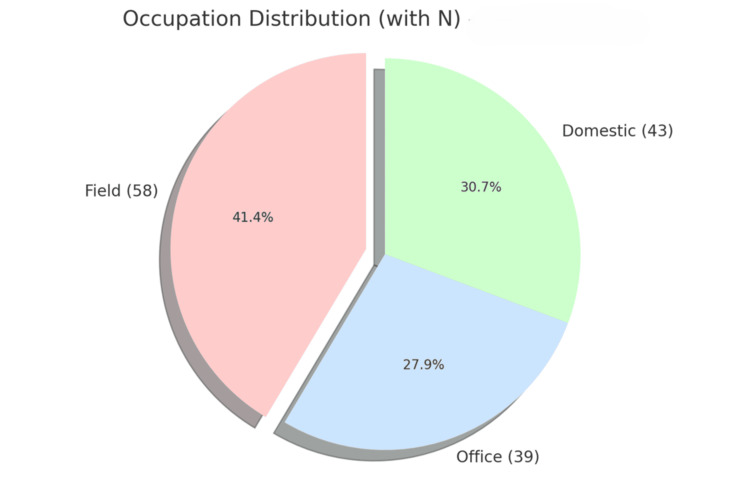
Occupation distribution among study participants

Figure 1 presents the distribution of individuals across three occupation categories: field, office, and domestic.

The relationship between various factors and the efficacy of PRP treatment for plantar fasciitis was evaluated (Table 2). It was found that age significantly influenced treatment outcomes, with patients aged 25-45 years demonstrating higher efficacy (91 out of 94, 96.81%) compared to those aged 46-65 years (40 out of 46, 86.96%), as indicated by a p-value of 0.026. In contrast, other factors such as gender, duration of disease, BMI, the affected side, occupation, and diabetes status did not significantly affect treatment efficacy.

**Table 2:** Stratification of efficacy with respect to the demographic profile of patients

Variable	Category	Efficacy, yes (n)	Efficacy, no (n)	p-value
Age (years)	25-45	91	3	0.026
	46-65	40	6	
Gender	Male	84	5	0.605
	Female	47	4	
Duration of disease	≤3 months	76	4	0.426
	>3 months	55	5	
BMI (kg/m²)	≤27	44	3	0.987
	>27	87	6	
Side affected	Right	65	3	0.344
	Left	66	6	
Occupation	Field	54	4	0.454
	Office	38	1	
	Domestic	39	4	
Diabetes mellitus	Yes	50	6	0.091
	No	81	3	

Both male (84 out of 89, 94.38%) and female (47 out of 51, 92.16%) patients showed similar responses to PRP treatment, with a p-value of 0.605. No significant differences in efficacy were observed based on the duration of symptoms, with 76 out of 80 patients (95.00%) showing improvement when the duration was three months or less, and 55 out of 60 patients (91.67%) improving when the duration was longer than three months (p = 0.426). Similarly, BMI did not significantly influence outcomes, as efficacy rates were 93.62% (44 out of 47) for patients with a BMI of 27 or less and 93.55% (87 out of 93) for those with a BMI above 27 (p = 0.987). The side of the body affected (right or left) also showed no meaningful impact on treatment efficacy, with 65 out of 68 patients (95.59%) experiencing improvement on the right side, and 66 out of 72 patients (91.67%) on the left side (p = 0.344). Among different occupational groups, efficacy rates were comparable, with efficacy seen in 54 out of 58 field workers (93.10%), 38 out of 39 office workers (97.44%), and in 39 out of 43 domestic workers (90.70%), with a p-value of 0.454. Although non-diabetic patients had a slightly higher efficacy rate (81 out of 84, 96.43%) compared to diabetic patients (50 out of 56, 89.29%), this difference was not statistically significant (p = 0.091).

Overall, age was the only factor that significantly impacted the efficacy of PRP treatment for plantar fasciitis, while other demographic and clinical variables did not show a meaningful influence.

## Discussion

Plantar fasciitis is a major cause of heel pain, responsible for 11% to 15% of foot disorders requiring medical intervention in adults. The condition is commonly linked to risk factors such as prolonged standing, obesity, and increased physical activity [11]. While plantar fasciitis typically affects individuals aged 40 to 60, there is no significant difference in prevalence between genders [3]. Diagnosis is usually clinical, and imaging is rarely required. First-line treatment procedures, including NSAIDs, cryotherapy, orthotics, stretching exercises, and shockwave therapy, are successful in over 90% of cases. However, about 10% of patients continue to experience symptoms and require more invasive treatment methods such as steroid injections, which carry risks such as plantar fascia rupture and infection [12-14].

Platelet-rich plasma has emerged as a potential alternative to corticosteroid injections for chronic plantar fasciitis, primarily due to its regenerative properties. PRP is rich in growth factors, which promote tissue repair and healing at a cellular level [15,16]. In our study, PRP showed a 93.57% success rate, consistent with the existing literature highlighting its efficacy. PRP delivers concentrated growth factors that address the degenerative changes seen in plantar fasciitis, promoting blood vessel formation and tissue repair in the plantar fascia [16].

When comparing PRP to corticosteroid injections, this study supports previous findings that PRP provides longer lasting pain relief and functional improvement. PRP results in significant long-term improvement in pain and daily activities, whereas corticosteroids tend to offer only short-term relief [12]. It has been emphasized that PRP not only provides longer lasting relief but also has fewer adverse effects, making it a safer long-term treatment option for plantar fasciitis [17].

However, PRP’s success largely depends on the method of preparation and injection technique. Studies suggest that using ultrasound guidance for PRP injections improves precision and outcomes, though our study did not employ ultrasound, which may have influenced the results [15]. PRP injections guided by ultrasound provide long-lasting pain relief with minimal complications, compared to repeated steroid injections [11]. Randomized trials also confirmed the superiority of PRP over corticosteroids in the long-term management of plantar fasciitis [18,19].

Despite promising results, variability in PRP preparation and injection techniques across studies highlights the need for standardized protocols. While this study, alongside others, supports the efficacy of PRP, larger controlled trials are required to establish clear guidelines for its use and to optimize patient outcomes.

Limitations

This study has several limitations. First, it was retrospective in nature and conducted at a single center, which introduces selection bias and limits the generalizability of the findings to a broader population. Second, the follow-up period of three months focuses only on short-term outcomes, and as a result, long-term efficacy and recurrence rates remain unclear. Third, the study lacks a control group or comparison group, such as patients receiving corticosteroid injections or undergoing surgery, which limits our ability to definitively conclude that PRP is superior to other treatment options.

Additionally, the variability in PRP preparation techniques and the absence of ultrasound guidance during injections may have influenced the outcomes. More consistent methods for PRP preparation and the use of ultrasound guidance could improve the accuracy of injections and potentially enhance patient outcomes.

## Conclusions

This study demonstrated that platelet-rich plasma is an effective treatment option for plantar fasciitis, with a high success rate in reducing pain and improving functional outcomes. PRP offers a minimally invasive alternative to conventional treatment methods like steroid injections, with fewer associated complications. Given these findings, PRP may be particularly beneficial for patients unresponsive to conservative treatment. However, further research, including larger, controlled trials with a long-term follow-up, is necessary to confirm these results and establish standardized guidelines for its use.
